# Unexpected Discovery of Hypermutator Phenotype Sounds the Alarm for Quality Control Strains

**DOI:** 10.1093/gbe/evab148

**Published:** 2021-06-28

**Authors:** Kun Wu, Zhou-Hua Cheng, Emily Williams, Nathan T Turner, Dapeng Ran, Haichao Li, Xia Zhou, Huilin Guo, Way Sung, Dong-Feng Liu, Michael Lynch, Hongan Long

**Affiliations:** 1 Institute of Evolution and Marine Biodiversity, KLMME, Ocean University of China, Qingdao, China; 2 Laboratory for Marine Biology and Biotechnology, Qingdao Pilot National Laboratory for Marine Science and Technology, Qingdao, China; 3 CAS Key Laboratory of Urban Pollutant Conversion, Department of Environmental Science and Engineering, University of Science and Technology of China, Hefei, China; 4 Center for Mechanisms of Evolution, The Biodesign Institute, Arizona State University, Tempe, Arizona, USA; 5 Department of Bioinformatics and Genomics, University of North Carolina, Charlotte, North Carolina, USA

**Keywords:** comparative genomics, DNA mismatch repair, genome evolution, *Shewanella*, mutation spectrum

## Abstract

Microbial strains with high genomic stability are particularly sought after for testing the quality of commercial microbiological products, such as biological media and antibiotics. Yet, using mutation–accumulation experiments and de novo assembled complete genomes based on Nanopore long-read sequencing, we find that the widely used quality-control strain *Shewanella putrefaciens* ATCC-8071, also a facultative pathogen, is a hypermutator, with a base-pair substitution mutation rate of 2.42 × 10^−8^ per nucleotide site per cell division, ∼146-fold greater than that of the wild-type strain CGMCC-1.6515. Using complementation experiments, we confirm that *mutL* dysfunction, which was a recent evolutionary event, is the cause for the high mutation rate of ATCC-8071. Further analyses also give insight into possible relationships between mutation and genome evolution in this important bacterium. This discovery of a well-known strain being a hypermutator necessitates screening the mutation rate of bacterial strains before any quality control or experiments.

## Introduction

Significance

We present one accidental discovery that the facultative pathogen *Shewanella putrefaciens* ATCC-8071, also widely used for biodegradation, biofuel, and quality control for microbiological products, is a natural hypermutator, due to the recent function-loss of the DNA mismatch repair gene *mutL*. Using de novo assemblies by Nanopore sequencing and ∼200 initially isogenic lines each accumulating spontaneous mutations for thousands of generations, this work also explores the association between mutation and genome evolution. This finding would hopefully become the theoretical basis for requiring microbiological stock centers to check the genomic stability of all their bacterial strains, before shipping to customers, as well as reminding researchers to always double-check the mutation rate of their strains before experiments.

Quality-control strains (QC strains) are used for evaluating the quality of commercial culture media and biochemical identification kits, and testing susceptibility of bacteria to antimicrobial agents (Lambert and Pearson 2000; Basu et al. 2005; Reller et al. 2009; Leclercq et al. 2013). High genomic stability of quality control strains is required for repeatable and reliable microbiological tests. For example, the minimal inhibitory concentration of certain antibiotics must be the same when used on a quality control bacterial strain before clinical therapy (Reller et al. 2009; Leclercq et al. 2013). A dramatically elevated mutation rate or genome instability could decrease the accuracy and repeatability of testing results.

Genomic instability is usually quantified by mutation rate of small-scale mutations, such as base-pair substitutions (BPSs) and indels, which are most abundant among mutations in DNA. Mutation is the ultimate source of evolution. However, too high a mutation rate may elevate the genetic load to a lethal level, and there are a wide variety of antagonizing mechanisms from molecular to population levels, such as DNA mismatch repair (MMR)—one of the most powerful DNA repair pathways in bacteria. Generally, the mutation rate in bacteria with functional DNA repair enzymes is about 0.001–0.003 mutation per genome per cell division, whereas there are also hypermutator bacteria, with orders of magnitude higher mutation rates than those of DNA-repair functional strains (Drake 1991; Oliver et al. 2000; Chopra et al. 2003; Foster 2007; Lee et al. 2012; Hammerstrom et al. 2015; Long, Sung, et al. 2015). The mutation rate of cells with MMR deficiency is greatly elevated in both prokaryotes and eukaryotes, especially in terms of transitions (Kunkel and Erie 2005). Most natural hypermutators result from the deficiency of MMR genes (Oliver et al. 2000; Chopra et al. 2003). Hypermutators are widely distributed in human environments. For example, 36% of patients with lung infections of *Pseudomonas aeruginosa* are colonized by mutator strains (Oliver et al. 2000). Approximately 25% *Helicobacter pylori* isolated from dyspeptic patients exhibit higher mutation frequencies than MMR defective Enterobacteriaceae (Bjorkholm et al. 2001).

*Shewanella putrefaciens*, a Gram-negative Shewanellaceae bacterium and widely found in marine environments, can degrade heavy metals, using Fe, U, and Tc as terminal electron acceptors during anaerobic respiration, that is, *S. putrefaciens* is a dissimilatory metal-reducing bacterium that can generate electricity by electron mediators—flavins—to deliver electrons. Recent studies also found that some strains of *S. putrefaciens* can result in human infection such as endocarditis (Dhawan et al. 1998; Holt et al. 2005) and multidrug resistance both in wild animals and clinical samples (Holt et al. 2005; Dias et al. 2018). The type strain of this bacterium—*S. putrefaciens* ATCC-8071—is also used as a quality-control strain for testing performance of antimicrobial agents, media, stains, and identification kits, as well as evaluating bacteriological procedures (Cherdtrakulkiat et al. 2016). Thus, from the human perspective, *S. putrefaciens* is a “two-faced” bacterium for being both pathogenic and beneficial.

In a broad investigation for mutation rates across the tree of life (Lynch et al. 2016; Long, Sung, et al. 2018), we accidentally discovered that one quality-control bacterium *S.putrefaciens* ATCC-8071, has a mutation rate about two orders of magnitude higher than most other DNA-mismatch-repair functional bacteria. Because its genome sequence and that of another wild-type control strain CGMCC-1.6515—a nonmodel and evolutionarily highly close strain, not experiencing repeated culturing to avoid the influence on mutation patterns by lab adaptation—have not been completed, de novo assembly was performed for both strains, using Oxford Nanopore long-read sequencing combined with Illumina PE150 sequencing. For both ATCC-8071 and CGMCC-1.6515, we also report the genomic mutation rates and mutation spectra from mutation accumulation (MA) experiments. The MA method applies repeated single-cell bottlenecks so that efficiency of selection is weakened such that most mutations have a high probability to be fixed in each line. We also explore the gene accounting for the elevated mutation rate of ATCC-8071, using complementation experiments. Comparative genomics and methylation analyses based on complete genomes of multiple *S. putrefaciens* strains were also done to clarify possible relationships between mutation and genome evolution in this important bacterium.

## Results

### High-Quality de novo Assemblies of *S. putrefaciens* ATCC-8071 and CGMCC-1.6515

**Fig. 1.**
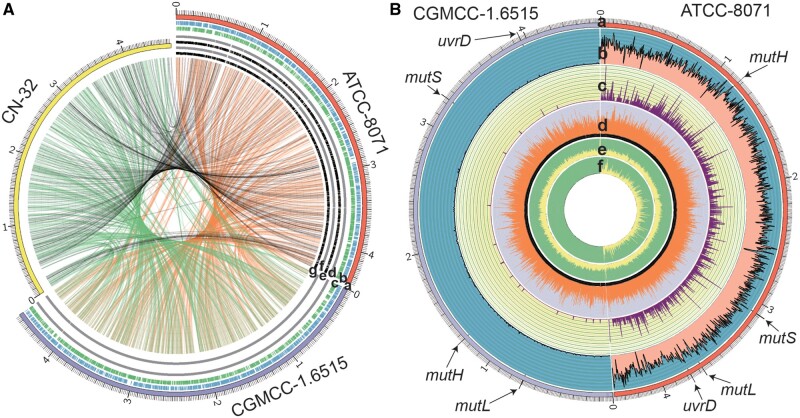
Summary of the genomic structure and mutation rate of ATCC-8071 and CGMCC-1.6515. (*A*) The genomic structure of ATCC-8071 and CGMCC-1.6515 and gene synteny of three strains. The outmost yellow, red, and purple curves with ruler lines represent the genome of CN-32, ATCC-8071, and CGMCC-1.6515, respectively. For the de novo assemblies of ATCC-8071 and CGMCC-1.6515, from the outside inward: genomic coordinates-labelled as a, coding sequences on plus strand-b, coding sequences on minus strand-c, methylated Dam target sites on plus strand-d, methylated Dam target sites on minus strand-e, methylated Dcm target sites on plus strand-f, methylated Dcm target sites on minus strand-g. Innermost circle shows synteny blocks (SBs) of genomic rearrangement events among three genomes of CN-32, ATCC-8071, and CGMCC-1.6515. (*B*) Distribution of mutation, expression level in FPKM, and methylation in the genomes of ATCC-8071 and CGMCC-1.6515. From the outside inward: the genomic coordinates-a, number of single nucleotide substitutions in 1,000 bins of genomes-b, number of indels in 1,000 bins of genomes-c, FPKM on each gene after log10 transformation-d, the number of *Dam* methylated sites in 1,000 bins of genomes-e and the number of Dcm methylated sites in 1000 bins of genomes-f. Four MMR genes are labelled for each genome.

Precise mutation analysis relies on high-quality reference genomes. However, there was no reference genome available for either *S. putrefaciens* ATCC-8071 or CGMCC-1.6515 that was used in this study. For *S. putrefaciens* ATCC-8071, we applied in-lab Nanopore sequencing technology to generate 2.59 Gb high-quality long reads (sequencing quality >10 and read length >10 kb). In order to improve the quality of the draft genome, 3.96-Gb Illumina PE150 clean reads were also generated using Illumina sequencing. Similarly, for CGMCC-1.6515, 1.68-Gb Nanopore high-quality long reads and 4.96-Gb Illumina clean reads were also generated. We used Unicycler (v-0.4.8) to assemble the genome, GenSAS (v-6.0) to annotate, and Pilon (v-1.23) to correct error bases, misassemblies, and fill gaps. The complete genomes of ATCC-8071 and CGMCC-1.6515 both contain one chromosome and no plasmid. The genome sizes for ATCC-8071 and CGMCC-1.6515 are 4,386,330 (GC content 44.39%) and 4,575,397 bp (GC content 47.02%), respectively (table 1 and fig. 1). We evaluated quality and completeness of the assemblies using QUAST (v-5.0.1) and BUSCO (v-2.0). Both genomes of ATCC-8071 and CGMCC-1.6515 are with high BUSCO scores (98.0 and 98.6, respectively). Other genomic details are listed in table 1 and figure 1*A* and *B*.

**Table 1:** Summary of Assemblies and Annotations of *Shewanella putrefaciens* ATCC-8071 and CGMCC-1.6515

Genomic Features	ATCC-8071	CGMCC-1.6515
Contigs	1	1
Largest contig	4,386,330	4,575,397
Total length	4,386,330	4,575,397
GC (%)	44.39	47.02
N50	4,386,330	4,575,397
N per 100 kb	0	0
BUSCO score	98	98.6
Replication origins	4,372,038–4,372,477	4,568,427–4,568,867
Replication terminus	2,144,755	2,287,501
Predicted genes	3,895	3,948
Number of rRNA operons	8	9
Number of tRNAs	101	105
Number of Dam motifs	15,321	16,976
Number of methylated Dam sites	27,602	30,380
Number of Dcm motifs	2,197	3,516
Number of methylated Dcm sites	4,338	29

Note.—*N*, the number of gaps in the assembly.

The origin of chromosomal replication (*oriC*) is crucial in regulating DNA replication and the cell cycle, and is associated with distribution of mutations and genome evolution. We identified *oriC* using Ori-Finder 2, which uses the distribution of Z-curves and DnaA box motif sequence homology with another closely related strain in the database—*S. putrefaciens* CN-32 (Luo et al. 2014; Luo et al. 2019). The origin of replication locates at 4,372,038–4,372,477 and 4,568,427–4,568,867 for ATCC-8071 and CGMCC-1.6515, respectively. The replication terminus is around 2,144,755 and 2,287,501, respectively, based on cumulative GC-skew plots.

There are 3,895 predicted protein-coding genes, 101 tRNAs, and eight rRNA operons in the genome of *S. putrefaciens* ATCC-8071 and 3,948 protein-coding genes, 105 tRNAs, and nine rRNA operons in the CGMCC-1.6515 genome (table 1). The gene numbers and gene-length distributions are highly similar to published *S.putrefaciens* genomes in the NCBI Genome database.

We also searched for prophage and CRISPR (Clustered Regularly Interspaced Short Palindromic Repeats) sequences in the genomes of the two strains. There are three incomplete prophage sequences with 7.53, 28.28 and 41.16 kb in length, and seven CRISPR sequences (two complete, five incomplete) in ATCC-8071. For CGMCC-1.6515, one incomplete 37.19 kb prophage and five CRISPR sequences (one complete, four incomplete) were detected (supplementary tables S1 and S2, Supplementary Material online).

In bacteria, DNA methylation plays an important role in protection from endonucleases, transcription, initiation of replication, and so on (Marinus and Lobner-Olesen 2014). N6-methyladenosines (m6A) and 5-methylcytosines (m5C) in DNA strands are known mutation hotspots (Barras and Marinus 1989; Holliday and Grigg 1993). The methylation mediated by m6A and m5C usually occurs at target motifs of DNA adenine methyltransferase (Dam) and DNA cytosine methyltransferase (Dcm), respectively, in bacteria. Nanopore sequencing can reveal methylated bases, which excite different electrolytic current signals from nonmethylated ones. We analyzed the methylation levels of Dam and Dcm target sites in both strands of the genome (Dam canonical target motif: 5′GATC3′, Dcm target motif: 5′CCWGG3′), using Tombo (v-1.5) (Stoiber et al. 2016) with Nanopore electrolytic current signals of *S. putrefaciens* ATCC-8071 and CGMCC-1.6515 as input. The most uncommon finding is that in CGMCC-1.6515, only 0.82% of Dcm target motifs are methylated (supplementary table S14, Supplementary Material online; table 1; fig. 1*A* and *B*), which is a sign of Dcm methyltransferase dysfunction. There is no *dcm* gene in the genome annotation either. To confirm that the lack of cytosine methylation in CGMCC-1.6515 is not an artifact of the analysis, but due to genetic background. We also blasted the *dcm* sequences of ATCC-8071 and CN-32 against the CGMCC-1.6515 genome. There is no hit and this further supports the lack of *dcm* in the CGMCC-1.6515 genome, and also confirms the efficacy of the methylation analysis based on Nanopore electrolytic current signals.

### Genome Evolution of *Shewanella* Species

**Fig. 2.**
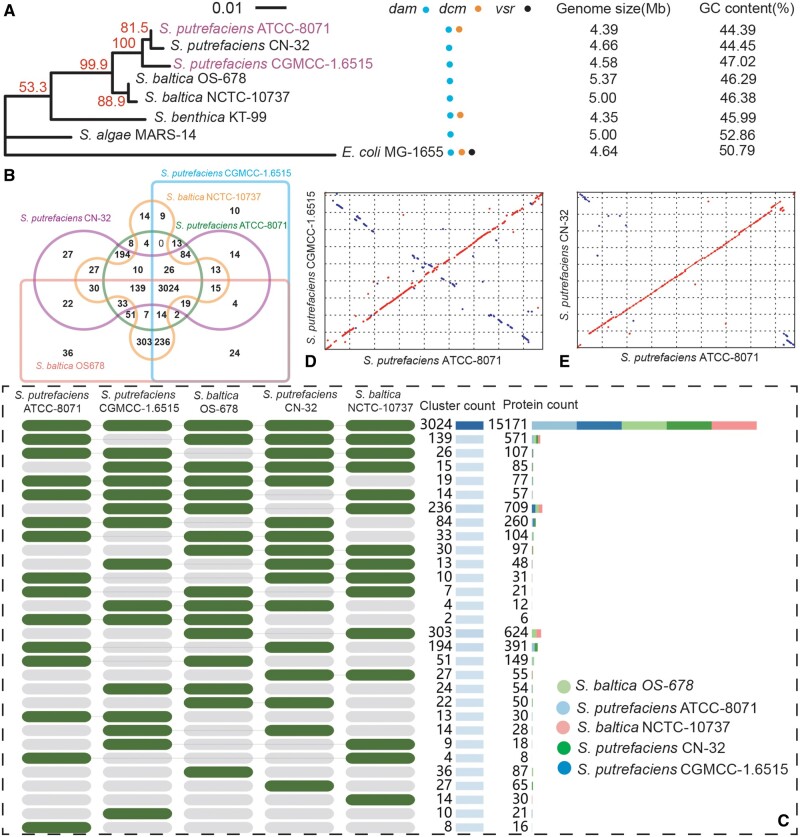
Genomic evolution among *Shewanella* species. (*A*) The maximum likelihood phylogenetic tree, evolution of methylation-associated genes, genome size, and GC content of eight strains. The phylogenetic tree is based on 16S rRNA of eight strains (*S. putrefaciens* CN-32, *S. baltica* NCTC-10737, *S. baltica* OS-678, *S. benthica* KT-99, and *S. algae* MARS-14 from NCBI, two of our assemblies *S. putrefaciens* ATCC-8071, *S. putrefaciens* CGMCC-1.6515, and *Escherichia coli* K-12 MG-1655 as the outgroup). The scale represents the number of substitutions and 1,000 bootstrappings were used. Colored dots refer to presence of the gene, based on genome annotation and BLAST. (*B*) Clusters of orthologous genes of five well-annotated *Shewanella* genomes (*S. putrefaciens* CN-32, *S. putrefaciens* ATCC-8071, *S. putrefaciens* CGMCC-1.6515, *S. baltica* NCTC-10737, and *S. baltica* OS-678). (*C*) The cluster counts and protein counts in the five genomes. (*D* and *E*) Dot matrices from genomic alignments of ATCC-8071 with CGMCC-1.6515 and CN-32, respectively. Red dots represent alignments in forward orientation. Blue plots represent alignments in reverse complement matches and these may be probably inverted repeats, or simply chance matches.

Orthologous genes are important playground for genome evolution. We construct the clusters of orthologous genes (OGs, and an OG cluster is defined as a gene cluster appearing in at least two species) from five *Shewanella* genomes that are completely assembled (*S. putrefaciens* ATCC-8071, *S. putrefaciens* CGMCC-1.6515, *S. baltica* OS-678, *S. putrefaciens* CN-32, and *S. baltica* NCTC-10737; fig. 2*A*); and their numbers of OGs clusters are 3,629, 3,508, 3,959, 3,681, and 3,871, respectively (supplementary table S3, Supplementary Material online). In total, 4,317 orthologous clusters from the five strains are identified (fig. 2*B* and *C*). There are 3,024 conserved OGs clusters (70.05%)—orthologous gene clusters present in all five genomes, which contribute to essential functions such as replication, transcription, translation, and metabolism. CN-32 shares the most OG clusters with ATCC-8071 that are absent in other strains (194 vs. 14, 27, 22 with CGMCC-1.6515, *S. baltica* NCTC-10737 and *S. baltica* OS-678, respectively; fig. 2*C*). There are only eight unique OG clusters in ATCC-8071, but 36, 14, 27, and 10 in *S. baltica* OS-678, *S. baltica* NCTC-10737, *S. putrefaciens* CN-32, and *S. putrefaciens* CGMCC-1.6515, respectively. This again shows the close evolutionary relationship between ATCC-8071 and CN-32, two strains widely used in *Shewanella* studies. ATCC-8071 does have 99 clusters that are not present in CN-32, the majority of which play important roles in various enzyme catalysis systems, including transporter activity, hydrolase activity, peptidase activity, and transferase activity.

Large-scale genome rearrangements such as duplication, deletion, insertion, inversion, and translocation are important drivers in genome evolution. To explore genome rearrangements of *S. putrefaciens*, we analyzed the syntenic relationships among the three complete genomes of ATCC-8071, CGMCC-1.6515, and CN-32. Based on Mummer (v-4.0.0beta2) (Kurtz et al. 2004), we find 33 and 44 genome rearrangements of ATCC-8071 and CGMCC-1.6515, respectively, in this analysis (supplementary tables S4 and S5, Supplementary Material online). CGMCC-1.6515 experienced a higher level of genome rearrangements (9.62 rearrangements per million base pairs) than ATCC-8071 (7.52 rearrangements per million base pairs) with CN-32 as the control for analyses, consistent with their phylogeny (figs. 1*A* and 2*A*).

We also find multiple inversions by aligning the genomes of ATCC-8071, CGMCC-1.6515, and CN-32 using the dot matrix function of Mummer. The dot matrices from ATCC-8071 versus CN-32 and ATCC-8071 versus CGMCC-1.6515 alignments both show X-shaped patterns and indicate multiinversions around the origins and termini of replication (fig. 2*D* and *E*). Previous studies found similar patterns to our results in other bacteria (Eisen et al. 2000; Suyama and Bork 2001) and this pattern may result from replication-directed translocation (Mackiewicz et al. 2001). The equidistant replication forks are always close to each other during replication and thus may lead to reciprocal recombination or translocation (Tillier and Collins 2000).

**Fig. 3.**
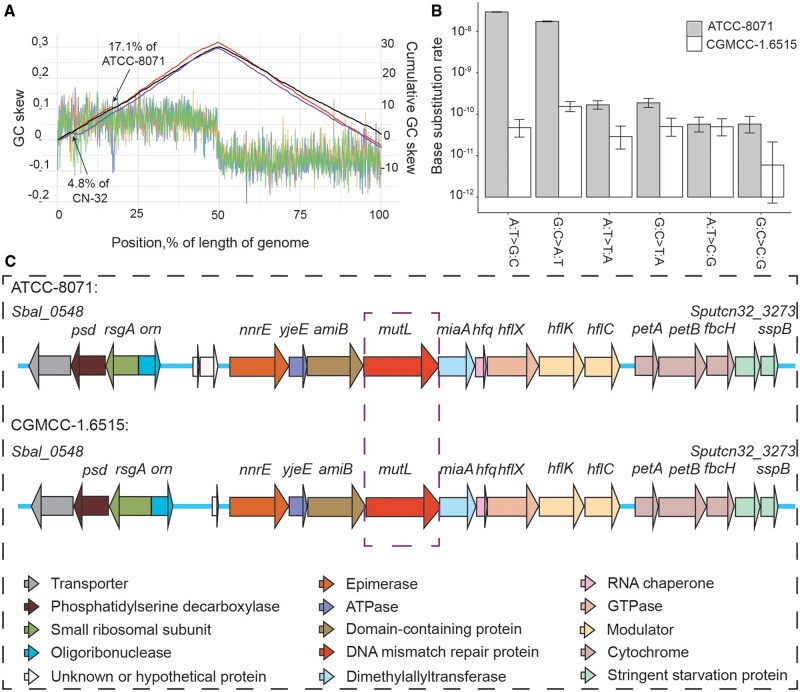
GC-skew, mutation spectra, and genes around *mutL* of *Shewanella putrefaciens* strains. (*A*) The GC-skew and cumulative GC-skew. The blue, orange, and green lines represent the distribution of GC-skew along the genomes of ATCC-8071, CGMCC-1.6515, and CN-32, which are equally divided into 1,000 bins, respectively. All genome positions start from the origin of replication. The red, black, and purple caret-shaped curves represent cumulative GC-skew in ATCC-8071, CGMCC-1.6515, and CN-32 genomes, respectively. GC skew is calculated by the formula: GC skew=(*G* − *C*)/(*G* + *C*), where *G* and *C* are the number of guanines and cytosines in each bin, respectively. (*B*) The mutation spectra of ATCC-8071 and CGMCC-1.6515. Base substitution rate is in the unit of per site per cell division. (*C*) Comparison of genes around *mutL* of *S. putrefaciens* of ATCC-8071 versus wild-type CGMCC-1.6515. Gene clusters near *mutL* are shown with arrows in different colors.

As inversions show opposite cumulative GC-skew trend from that of noninversion regions in the same replichore, we also detect dozens of inversion events in the *S. putrefaciens* three strains, using the cumulative GC-skew method (fig. 3*A* and supplementary tables S6–S8, Supplementary Material online). Most notably, there are two large inversions (>25 kb) in the 731,329–757,645 position of ATCC-8071 and 174,330–211,602 of CN-32 genomes, involving 27 and 40 genes, respectively (supplementary tables S9 and S10, Supplementary Material online).

### Mutation Rate and Spectrum of *S. putrefaciens*

Mutation fuels genome evolution. In order to find out the role of mutation in shaping *S. putrefaciens* genome architecture, we first performed fluctuation tests to preliminarily investigate mutation rates. The mutation rate of ATCC-8071 (7.81 × 10^−8^, 95% CI: 5.62 × 10^−8^, 9.99 × 10^−8^; measured on the subculture strain CGMCC-1.3667) is 67.91 and 61.02 times higher than CGMCC-1.6515 (1.15 × 10^−9^, 95% CI: 7.10 × 10^−10^, 1.60 × 10^−9^) and CN-32 (1.28 × 10^−9^, 95% CI: 2.51 × 10^−10^, 2.31 × 10^−9^), strains with functional MMR, respectively.

In order to reveal the genomic mutation rate and spectrum, we then ran mutation accumulation (MA) experiments—the most accurate method for mutation rate estimation to date—on ATCC-8071 and CGMCC-1.6515. 76 and 110 MA lines were initiated, with each line experiencing 3,991 and 1,553 cell divisions on average, for ATCC-8071 and CGMCC-1.6515, respectively. All lines were sequenced with Illumina PE150 mode (Hiseq2500 for ATCC-8071 and X-10 for CGMCC-1.6515). About 45 and 102 lines are used in the final mutation analyses, after we filtered out lines that failed in library construction, genome sequencing (<15× depth of coverage), and/or cross contamination.

For ATCC-8071, the 45 mutation accumulation lines were sequenced to mean depth of coverage 91× (ranging from 16 to 128×) and mean mapping rate of 97.90% (SD = 0.006). Each MA line accumulated 420 BPSs on average (ranging from 261 to 772). In total, we identified 18,921 BPSs across all 45 MA lines, yielding a BPS mutation rate of 2.42 × 10^−8^ per nucleotide site per cell division (95% Poisson CI: 2.39 × 10^−8^, 2.46 × 10^−8^), with a transition/transversion ratio of 102.39. BPSs distributed along the genome in a symmetrical wave-like pattern around the origin of replication (supplementary fig. S1, Supplementary Material online). The extremely high transition/transversion ratio and the mutation spectrum are highly similar to those of the MMR-dysfunctional bacterial hypermutators previously reported (Foster et al. 2013; Long, Sung, et al. 2015) (fig. 3*B*; tables 2 and 3). The mutation rate in the GC direction and that in the AT direction yield a mutation bias to GC of 1.68—a characteristic GC-bias in many MMR-dysfunctional bacteria (Lee et al. 2012; Long, Sung, et al. 2015), implying an expected equilibrium GC content of 0.63 in the absence of selection, higher than the GC content 0.48 at 4-fold degenerate sites of the genome. We also detect 1,127 small indels (insertions/deletions), which lead to an indel mutation rate of 1.44 × 10^−9^ per site per cell division (95% CI: 1.36 × 10^−9^, 1.53 × 10^−9^). The ratio of insertion to deletion is 1.41 and 96.10% of indels occur in simple sequence repeat (SSR) regions (supplementary tables S17–S19, Supplementary Material online and fig. 1*B*), that is, an insertion bias, again, highly similar to reported MMR-dysfunctional strains.

**Table 2:** The Mutation Spectra of *Shewanella putrefaciens* ATCC-8071 and CGMCC-1.6515

	ATCC-8071	CGMCC-1.6515
Categories	Count/Proportion	Count/Proportion
Intergenic regions	2,169/0.11	27/0.23
Coding regions	16,752/0.89	92/0.77
Overlap	13/7.76 × 10^−4^	0/0
Synonymous	6,190/0.37	31/0.34
Nonsynonymous	10,549/0.63	61/0.66
Transitions	18,738/0.99	70/0.59
A:T→G:C	12,739/0.68	18/0.26
G:C→A:T	5,999/0.32	52/0.74
Transversions	183/0.01	49/0.41
A:T→T:A	73/0.40	11/0.22
A:T→C:G	25/0.14	19/0.39
G:C→C:G	20/0.11	2/0.04
G:C→T:A	65/0.36	17/0.35
Insertions	660/0.59	5/0.29
Deletions	467/0.41	12/0.71

Note.—Nonsynonymous, base-pair substitutions causing amino acid change; synonymous, mutations not causing amino acid change; overlap, mutations occurring in overlapped reading frames; count, the total number of mutations across all MA lines; proportion, the proportion of the mutations in the category out of the total BPS/indel mutations across all MA lines—intergenic versus coding, transitions versus transversions, insertions versus deletions.

**Table 3:** Counts and Proportions of Different BPSs/SNPs from Different Datasets

Datasets	BPSs/SNPs	Count	Proportion
ATCC-8071	GC ↔ AT	1,916	0.62
ATCC-8071	CG ↔ AT	585	0.19
ATCC-8071	AT ↔ TA	301	0.10
ATCC-8071	GC ↔ CG	264	0.09
CGMCC-1.6515	GC ↔ AT	28,288	0.54
CGMCC-1.6515	CG ↔ AT	12,623	0.24
CGMCC-1.6515	GC ↔ CG	5,943	0.11
CGMCC-1.6515	AT ↔ TA	5,759	0.11
ATCC-8071_MA	GC ↔ AT	18,738	0.99
ATCC-8071_MA	CG ↔ AT	90	0.005
ATCC-8071_MA	AT ↔ TA	73	0.004
ATCC-8071_MA	GC ↔ CG	20	0.001
CGMCC-1.6515_MA	GC ↔ AT	70	0.59
CGMCC-1.6515_MA	CG ↔ AT	36	0.30
CGMCC-1.6515_MA	AT ↔ TA	11	0.09
CGMCC-1.6515_MA	GC ↔ CG	2	0.02

Note.—ATCC-8071, natural SNPs in the genome; ATCC-8071_MA, BPSs from MA lines; CGMCC-1.6515, natural SNPs in the genome; CGMCC-1.6515_MA, BPSs from MA lines; Count, the total number of BPSs/SNPs in the MA lines/genome of the strain; proportion, the proportion of the BPSs/SNPs in the category out of the total BPS/SNPs in the MA lines/genome of the strain.

For CGMCC-1.6515, we detected 119 BPSs from 102 MA lines with mean coverage of 155× (ranging from 72 to 423×) and mean mapping rate of 98.03% (SD = 0.012), yielding a BPS mutation rate of 1.66 × 10^−10^ per nucleotide site per cell division (95% Poisson CI: 1.38 × 10^−10^, 2.00 × 10^−10^), almost identical with the mutation rate observed in *E. coli* MA experiments (Lee et al. 2012). The transition/transversion ratio is 1.43. The mutation bias to GC is 0.48 and indicates a slight AT mutation bias, a pattern found in most bacteria. The GC content expected from mutation alone (0.32) is lower than the GC content at 4-fold degenerate sites of the genome (0.58), consistent with the idea of GC selection at silent sites in bacteria (Long, Sung, et al. 2018). In CGMCC-1.6515, we find five insertions and 12 deletions, yielding an insertion/deletion ratio of 0.42 and an indel mutation rate of 2.39 × 10^−11^ (95% Poisson CI: 1.39 × 10^−11^, 3.82 × 10^−11^), and consistent with wild-type bacterial indel rates being ∼5–10× lower than the base-substitution mutation rates (Sung et al. 2016). 88.24% of the indels are in SSR motifs (supplementary tables S20–S22, Supplementary Material online and fig. 1*B*). When compared with ATCC-8071, the BPS and indel mutation rates are 145.78× and 60.25× higher than those of CGMCC-1.6515, respectively. The highly elevated transition/transversion ratio and high insertion/deletion ratio in ATCC-8071 are also phenotypes of MMR deficiency, since MMR preferentially repairs transitions and insertions (Long, Miller, et al. 2018). Taken together, ATCC-8071, the type strain of *S. putrefaciens* and quality-control strain widely used in microbiology research and industry, appears to be a hypermutator caused by MMR-dysfunction. The genome GC content 44.39% of ATCC-8071 is lower than 47.02% of the wild-type CGMCC-1.6515 (table 1), inferring that ATCC-8071 might not be a long-term mutator, of which GC content would have been elevated by its mutation bias to the GC direction.

We also explore the association between gene length and mutation number, using mutations accumulated in ATCC-8071 genes (supplementary table S23, Supplementary Material online). Longer genes do have a higher number of mutations due to larger mutational space (Pearson’s correlation test, *r *=* *0.80, *P *<* *2.2 × 10^−16^). Among them, the 4,449-bp *gltB* (glutamate synthase, large subunit) is the only gene showing a mutation rate significantly higher than the genomic mutation rate (8.74 × 10^−8^ per site per cell division vs. 2.42 × 10^−8^; Poisson test with Bonferroni correction, *P *=* *5.45 × 10^−6^; supplementary table S23, Supplementary Material online). *gltB* is involved in nitrogen metabolism and codes for a subunit of glutamate synthase, which catalyzes glutamate biosynthesis (Miller and Stadtman 1972). We tested selection, methylation, and nucleotide context, whereas none could explain the high mutation rate. Then, could transcription associated mutagenesis be the cause? Transcription requires unwinding of DNA double strands and the process would topologically generate torsional stress, sensitize the loose single strand when exposed to damaging agents and supercoil the upstream and downstream of transcription bubble especially in longer genes (Jinks-Robertson and Bhagwat 2014; Thompson et al. 2020). We thus performed RNAseq of ATCC-8071 and analyzed the transcription profile. The product of transcription level (measured in Fragments Per Kilobase of transcript, per million mapped reads—FPKM) and gene length could reflect the total frequency of single-strand exposure during transcription. Such product of *gltB* is at the top 14.17% among those of all genes (supplementary table S23, Supplementary Material online). We also find the number of *gltB* mutations is highly positively correlated with nucleotide position (Pearson’s correlation test, *r *=* *0.89, *P *=* *0.041, top 1.90% in all genes), with more mutations present in the downstream of *gltB* (4, 6, 11, 9, 12 in the first, second, third, fourth, and last 20% of *gltB*). Thus, the extremely high mutation rate of *gltB* might result from the synergistic effect of high transcription frequency and RNA polymerase replication fidelity decrease in longer genes (supplementary table S23, Supplementary Material online).

We did not detect any mutations in noncoding rRNA operons. These operons usually consist of promoter, 5S rRNA, 16S rRNA, tRNA, 23S rRNA, and terminator (vs. 19.73—expected number of mutations in 5S rRNA + 16S rRNA + 23S rRNA across all final MA lines; Poisson test, *P* ∼ 0) (Apirion and Miczak 1993). There are eight rRNA operons in the ATCC-8071 genome and all genes in the rRNA operons are covered with high-coverage reads. This is consistent with rRNA homologues being almost identical in most other bacteria (Liao 2000). In order to further confirm that no-mutation hit in rRNA operons is a general pattern among bacteria, we performed local mutation analysis of rRNA operons in another three MMR-deficient bacteria studied with MA: *Escherichia coli* K-12 MG1655 *ΔmutS*, *Pseudomonas fluorescens* SBW25 *ΔmutL*, and *Vibrio fischeri* ES114 *ΔmutS* (supplementary table S24, Supplementary Material online) (Long et al. 2016; Dillon et al. 2017; Long, Miller, et al. 2018). Indeed, there are no mutations detected and this indicates that high conservation in rRNA operons exists in both long-term evolution and short-term mutation accumulation experiments. We speculate that this results from either the functional constraints of ribosomes, or concerted evolution of rRNA homologs with gene conversion involved. rRNA operons with zero mutations detected should not be explained by functional constraints or selection, since not all rRNA operons are indispensable. For example, *E. coli* can still survive even with only one of its seven rRNA operons or with rRNA operons being replaced by exogenous operons from other strains (Asai, Condon, et al. 1999; Asai, Zaporojets, et al. 1999). Concerted evolution seems to be more reasonable (Liao 2000; Espejo and Plaza 2018), during which any mutation occurring in one rRNA operon is converted back to the majority base by the nonreciprocal recombination with double-stranded break repair and synthesis-dependent strand annealing involved (Orr-Weaver et al. 1988; Hastings 2010).

### Cause for the Hypermutator Phenotype in *S. putrefaciens* ATCC-8071

Compared with CGMCC-1.6515, the mutational features of ATCC-8071 support that its extremely high mutation rate originates from MMR dysfunction. The gene orders around MMR in the two strains are the same (fig. 3*C*). In order to find out which gene in the MMR system accounts for the mutation rate elevation, we introduced *mutS*, *mutL*, and *mutH* of CGMCC-1.6515 separately into ATCC-8071 cells by transformation of the reconstructed pYYDT plasmids, and performed fluctuation tests to estimate the mutation rates of the constructed strains. The mutation rate of ATCC-8071 and ATCC-8071 with *mutS*, *mutL*, and *mutH* of CGMCC-1.6515 is 7.92 × 10^−8^ (95% CI: 5.62 × 10^−8^, 1.02 × 10^−7^), 6.78 × 10^−8^ (4.95 × 10^−8^, 8.61 × 10^−8^), 3.16 × 10^−9^ (1.50 × 10^−9^, 4.82 × 10^−9^), and 7.91 × 10^−8^ (5.88 × 10^−8^, 9.94 × 10^−8^), respectively (supplementary fig. S5, Supplementary Material online). Only does the mutation rate of ATCC-8071::pYYDT-*mutL* show dramatic decrease to a level, which is not significantly different from that of CGMCC-1.6515 (1.15 × 10^−9^, 95% CI: 7.10 × 10^−10^, 1.60 × 10^−9^). All these results show that hypermutation rate of ATCC-8071 is caused by the dysfunction of *mutL*. In order to find out the exact molecular defects of ATCC-8071’s *mutL*, we aligned the *mutLs* of ATCC-8071 and CGMCC-1.6515 at both the DNA and amino acid levels (supplementary fig. S6, Supplementary Material online). However, the DNA sequence similarity is quite low (Levenshtein distance 77.95%); there are 111 replacements and six indels at the amino acid level. Extensive gene editing is needed to identify the specific mutation(s) leading to dysfunction of the ATCC-8071 *mutL* in the future.

### BPSs in MA Lines versus Natural SNPs in the Hypermutator and Wild-Type Strains

In MA experiments, the effective population size is extremely low and mutations experience much weaker selection than strains in nature. Thus, comparing BPSs of CGMCC-1.6515 and ATCC-8071 MA lines with SNPs accumulated in their genomes helps reveal the roles of mutation and natural selection in genome evolution. We aligned the genomes of three *S. putrefaciens* strains (ATCC-8071, CGMCC-1.6515, CN-32) and parsed out the biallelic SNPs at 4-fold degenerate sites present in ATCC-8071 and CGMCC-1.6515. We then collapsed the mutations from MA and SNPs from six mutation types to four, since mutation direction could not be determined in SNPs without knowing the true ancestral state.

In ATCC-8071 and CGMCC-1.6515, transitions in BPSs of MA or natural SNPs dominate transversions, as known in most other organisms (table 3). Specifically, G:C ↔ A:T BPSs/SNPs are more abundant than the sum of A:T ↔ T:A, C:G ↔ A:T, and G:C ↔ C:G transversions, making up ∼62%, 99%, 54%, and 59% of all BPSs/SNPs in ATCC-8071, ATCC-8071_MA, CGMCC-1.6515, and CGMCC-1.6515_MA, respectively. In the wild-type CGMCC-1.6515, the rankings of different natural SNPs are highly consistent with those of BPSs from MA, implying that mutations play important roles in shaping the genome content. Considering the elevated GC content at 4-fold degenerate sites as mentioned above (4-fold degenerate site GC content 0.58 vs. equilibrium GC content 0.32 based on mutation pressure alone), selection and/or gene conversion in favor of G:C are also critical in *S.putrefaciens* genome evolution, similar to most organisms recently studied (Long, Sung, et al. 2018).

Interestingly, in the hypermutator ATCC-8071, the natural SNP spectrum closely resembles that of CGMCC-1.6515 (table 3). Although its expected equilibrium GC content 0.63 based on mutation bias is higher than 0.48—the GC content at 4-fold degenerate sites. This is highly unusual in studied organisms, since selection and/or gene conversion is known to elevate GC content at 4-fold degenerate sites (Long, Sung, et al. 2018). One possible explanation is that ATCC-8071 has lost its repair function quite recently—not a long-term hypermutator, and the genome has not reached mutation–selection equilibrium.

## Discussion

ATCC-8071—the type strain of *S.putrefaciens*—is widely used as a quality-control strain. For example, it is used in Culti-Loops to test performance of media, stains, reagents, and identification kits. Thus, genomic stability for such a strain is crucial for reliable and repeatable microbiology products quality control. Our discovery of the natural hypermutator strain ATCC-8071 is a critical finding, as this is the first reported hypermutator strain of the model bacterium *S.putrefaciens*, and furthermore informs clinicians and researchers that this strain may not be as stable as originally thought. Except for natural mutators, laboratory-evolved bacteria may also contribute to the hypermutation phenotype (Liu et al. 2017; Desroches et al. 2018). This study provides a clear case, demonstrating for the urgent need to check strains for the hypermutator phenotypes prior to research and/or testing.

Comparative genomics shows large divergence in genome architecture of the hypermutator strain ATCC-8071 from other closely related wild-type strains in properties such as the amount of prophages, CRISPR sequences, and general genomic synteny. Variation in the genome structure might be resulted from elevated mutation rates, especially that of structural variants (Modrich and Lahue 1996). Unfortunately, due to the short-read sequencing technologies that we used in this study, we were unable to detect any reliable structural variants in our MA lines.

As revealed by the BPS mutations from wild-type CGMCC-1.6515 and hypermutator ATCC-8071 MA lines of *S.putrefaciens*, the genome-wide mutation rates and spectra are, respectively, highly similar to other wild-type and hypermutator bacteria studied (Long, Miller, et al. 2018). In the wild-type strain, we observe that evolutionary forces such as selection is operating to elevate the GC nucleotide composition at 4-fold degenerate sites. However, the hypermutator strain shows a different pattern of genome evolution, that is, GC content at 4-fold degenerate sites is lower than the expected GC content by mutation pressure alone. This suggests that hypermutators are evolving differently than the wild-type strains and places further importance on verifying mutation rates of focal strains prior to study.

Methylation also plays important role in genome evolution. Dam participates in various functional processes such as MMR repair, replication initiation, gene expression, transcriptional regulation, and so on. The lack of Dam may lead to a lethal consequence, but there is no lethality observed for dysfunction of Dcm (Marinus and Lobner-Olesen 2014; Sanchez-Romero et al. 2015). Previous studies propose that *dam* genes are present in various bacteria, whereas *dcm* genes only exist in the members of Enterobacteriaceae (Palmer and Marinus 1994; Marinus and Lobner-Olesen 2014). In *Shewanella* species (Shewanellaceae), Dam is present in all species, and Dcm also exists in *S. benthica* KT-99 and *S. putrefaciens* ATCC-8071, though *S. putrefaciens* CGMCC-1.6515 does not have a *dcm* gene as predicted (fig. 2*A*). Methylated-cytosine deamination in Dcm target motifs leads to T:G mismatches and *vsr* (Very Short patch Repair) is usually involved in their repair (Lieb and Bhagwat 1996; Marinus and Lobner-Olesen 2014). Interestingly, we did not find *vsr* in any *Shewanella* species, inferring that other pathways might function for repairing T:G mismatches in Dcm target sites. Taken together, Dam and Dcm receive different levels of selection, which might have driven the contrasting sporadic and universal distribution of Dcm and Dam in bacteria, respectively, and further promotes genome divergence. The unexpected presence of Dcm in some *Shewanella* species invokes the need for more exploration on Dcm distribution and function in more bacteria species, especially those with complete genomes, which would eventually give a true picture of the role of methylation in the evolutionary process.

In-lab Nanopore sequencing in this study is demonstrated to be convenient, powerful, and reliable in de novo genome assembling, as well as sensitive and specific in detecting methylated bases. As its price decreases and accuracy increases, this technology will allow for more exciting studies, especially revealing the relationship between methylation and genome evolution.

## Materials and Methods

### Strains and Cultures

*Shewanella putrefaciens* ATCC-8071 was ordered from the American Type Culture Collection (ATCC), Inc., for mutation accumulation. Another equivalent strain CGMCC-1.3667—a strain recently propagated from ATCC-8071—was later ordered from China General Microbiological Culture Collection (CGMCC) for de novo assembling the ATCC-8071 genome and also for testing if the hypermutation rate detected from MA of ATCC-8071 was an artifact during subculturing using fluctuation tests, as it is not uncommon for lab-cultured bacterial strains (Desroches et al. 2018). We also ordered another two strains, *S. putrefaciens* CGMCC-1.6515 from CGMCC (collected from a cold water spring 23 meters underground of Inner Mongolia, China) and *S. putrefaciens* CN-32 ordered from ATCC (ATCC BAA-1097) as wild-type controls for mutation rate comparison with ATCC-8071 used in MA or fluctuation tests. Trypticase soy agar (BD Bacto 236950) or trypticase soy broth (BD Bacto 211825) were used for cell culturing during MA lines transfer, fluctuation tests, freezing, and DNA extraction.

### MA Transfers and Sequencing

About 76 and 110 MA lines were initiated from a single colony of *S. putrefaciens* ATCC-8071 and CGMCC-1.6515, respectively. Each line was single-colony transferred daily on trypticase soy agar at 26 °C. The cell divisions between two adjacent transfers (or one culturing cycle from a single cell to a colony) were estimated through colony forming units (CFU) every 30 days, and the grand mean of these estimates for cell divisions during each culturing cycle lead to ∼27 and 26 cell divisions, respectively, in ATCC-8071 and CGMCC-1.6515, across the entire experiments. Eventually, each MA line went through 3,991 and 1,553 cell divisions, or 148 and 60 single-colony transfers for ATCC-8071 and CGMCC-1.6515, respectively. The genomic DNA of all MA lines was extracted with Wizard Genomic DNA Purification Kit (Promega, Madison, WI).

DNA libraries for ATCC-8071 MA lines were constructed using the Nextera DNA Sample Prep Kit (Illumina, Inc.) with an insert size of 300 bp and an Illumina Hiseq2500 sequencer was used for PE150 sequencing at Hubbard Center for Genome Studies, University of New Hampshire. We constructed the DNA libraries of CGMCC-1.6515 MA lines, using a modified protocol for NEBNext Ultra II FS DNA Library Prep Kit for Illumina and Illumina PE150 sequencing was performed on an X-10 machine of Berry Genomics, Beijing (Li et al. 2019).

### Genome Sequencing, de novo Assembling, and Annotation of *S. putrefaciens* ATCC-8071 and CGMCC-1.6515

The genomic DNA of *S.putrefaciens* ATCC-8071 and CGMCC-1.6515 for de novo assembly was extracted with Qiagen MagAttract HMW DNA Kit (Cat. No. 67563) and quantified using a Qubit 3.0 fluorometer, library was constructed by the Ligation Sequencing Kit of Oxford Nanopore technology (SQK-LSK109), and sequencing was done using MinION (Flow cells R9.4) in the lab (for ATCC-8071) and by Nextomics Biosciences, Wuhan, China (for CGMCC-1.6515). In order for error correction and gap filling, Illumina PE150 sequencing was also performed with a Novaseq 6000 sequencer by Berry Genomics, Beijing.

For ATCC-8071, Nanopore sequencing yielded 444,000 raw reads, ∼7.05 Gb with the longest read of 177,590 bases; and for CGMCC-1.6515, 196,823 raw reads, or ∼3.22 Gb with the longest read of 122,376 bases. We then filtered out low quality (quality score <10) and short reads (length <10,000 bp) by NanoFilt (De Coster et al. 2018). After filtering, 2.59 and 1.68 Gb of Nanopore reads along with 3.96 and 4.96 Gbp clean Illumina short reads were retained for assembly of ATCC-8071 and CGMCC-1.6515, respectively. The de novo assembling of *S.putrefaciens* ATCC-8071 and CGMCC-1.6515 was done with Unicycler (v-0.4.8) (Wick et al. 2017), using both Nanopore long reads and Illumina short reads. Then we corrected error bases, misassemblies, and filled gaps with Pilon (v-1.23) (Walker et al. 2014). The quality of assemblies was evaluated by QUAST (v-5.0.1), BUSCO (v-2.0), and syntenic analysis (see below) (Simão et al. 2015; Mikheenko et al. 2018). We also searched for antibiotic resistance genes using the Comprehensive Antibiotic Resistance Database (CARD) (Alcock et al. 2020). CRISPR sequences were detected using CRISPRFinder (Grissa et al. 2007). PHAST based on glimmer (v-3.02) was used for searching prophages in the genomes (Zhou et al. 2011).

For RNAseq of ATCC-8071 and CGMCC-1.6515, colonies were grown at the same condition as MA. RNA was then extracted using Epicentre MasterPure Complete DNA and RNA Purification Kit (Cat. No.: MC85200). In addition, the concentration of RNA was quantified via a Qubit 3.0 fluorometer and purity measured using a microvolume spectrophotometer instrument (nano-300). cDNA library was constructed by Ribo-off rRNA Depletion Kit for bacteria (Vazyme, Cat. N407-02), VAHTS mRNA-seq V3 Library Prep Kit for Illumina (NR611-01), and Illumina PE150 sequencing was performed by a Novaseq 6000 sequencer of Berry Genomics, Beijing, eventually yielding 8.66 Gb and 7.31 Gb clean reads for ATCC-8071 and CGMCC-1.6515, respectively.

Transcriptome assembling was carried out with Trinity (v-2.8.5) using default parameters and structural prediction was done with Genemarks (v-3.36) on GenSAS (v-6.0) online platform (Besemer et al. 2001; Grabherr et al. 2011; Humann et al. 2019). Functional annotation was done using OmicsBox (v-1.1.78) and tRNA and rRNA were identified by tRNAscan-SE (v-2.0) (Lowe and Chan 2016; Chan and Lowe 2019; Chan et al. 2019) and barrnap (v-0.9) (https://github.com/tseemann/barrnap), respectively. For the estimation of FPKM, RNAseq reads were first aligned by Bowtie (v-2.2.9) (Langmead and Salzberg 2012) and then expression levels were analyzed using Cufflinks (v-2.2.1) (Trapnell et al. 2010).

### Mutation Analyses

We followed the method by Long et al. (2018). Briefly, we required at least 15× depth of coverage and no cross-line contamination. This eventually led to 45 and 102 MA lines for ATCC-8071 and CGMCC-1.6515, respectively, in the final analyses. After trimming adaptors by Trimmomatic (v-0.36) (Bolger et al. 2014), the trimmed reads of each MA line were mapped to the reference genome with Burrows-Wheeler Aligner (v-0.7.17) mem (Li and Durbin 2009). The HaplotypeCaller module in Genome Analysis Toolkit (GATK v-4.1.2) was used for calling SNPs and short indels using GATK’s best practices recommendations and only unique mutations were considered (McKenna et al. 2010; DePristo et al. 2011; Van der Auwera et al. 2013). Validation of SNPs and indels with Integrative Genomics Viewer (IGV) was also performed (Thorvaldsdóttir et al. 2013). Pooled mean mutation rate µ was calculated by µ$=\frac{m}{\sum_{1}^{n}N\times T}$, where *m* is the total number of mutations fromall MA lines, *n* is the total number of MA lines, *N* is the analyzed sites in each line, and *T* is the number of cell divisions each MA line passed. Standard error of the mean mutation rate was calculated by SEM=$\;\frac{SD}{\sqrt{n}}$, where SD is the standard deviation of the line-specific mutation rates. The CIs of mutation-rate estimates were calculated using the Poisson cumulative distribution function approximated by the $\;\chi^{2}\;$ distribution (Long, Kucukyildirim, et al. 2015). Context-dependent mutation rate is calculated using pipelines developed in Long, Sung, et al. (2015). Equilibrium GC content was calculated by *P*=$\frac{\mu }{\mu +v}$, where *μ* is the mutation rate in the GC direction (the sum of the A:T→G:C transition rate and the A:T→C:G transversion rate), and *v* is the mutation rate in the AT direction (the sum of the G:C→A:T transition rate and the G:C→T:A transversion rate) (Long, Sung, et al. 2018).

### Methylated Sites Identification

Tombo (Stoiber et al. 2016) was used to analyze the methylated sites, we first performed base calling with guppy (ONT developer access required, v-2.1.3), and converted muti-fast5 files to single-fast5 files using ont-fast5-api (v-1.4.0) with default parameters (https://github.com/nanoporetech/ont_fast5_api). After annotating single-fast5 files with fastq files and resquiggling, we used Specific Alternate Base Detection model to detect methylated sites, including Dam and Dcm methylated sites. This model computes a statistic similar to a log likelihood ratio (LLR) to identify the sites where signal matched the expected levels for an alternate base better than the canonical expected levels. We defined methylated sites as those with dampened fraction >0.9 (which represented the ratio of methylated signal to unmethylated).

### Fluctuation Tests

The mutation rate estimation using fluctuation tests was based on the Lea–Coulson model, which has 11 assumptions (Lea and Coulson 1949). Cells from a single colony of *S. putrefaciens* CGMCC-1.3667 or CGMCC-1.6515 were diluted in 1× PBS buffer (10 mM). ∼200 cells were inoculated into each of the 19 test tubes with 3 ml trypticase soy broth and incubated for 17 h until OD = 1 at 26 °C. Cell density was estimated by serially diluting 100 μl liquid culture and counting colony forming units. 1 ml liquid culture from each test tube was concentrated to 100 μl by centrifuging, plated onto trypticase soy agar containing 100 µg/ml rifampicin, and incubated at 26 °C for 36 h (Long, Sung, et al. 2015). Mutation rates and confident intervals were calculated using bz-rates (Gillet-Markowska et al. 2015).

### Phylogenetic Tree Construction and Syntenic Analysis

For the 16S rRNA-based phylogenetic tree, we used 16S rRNA sequences of seven *Shewanella* strains (*S. putrefaciens* CN-32, *S. putrefaciens* ATCC-8071, *S. putrefaciens* CGMCC-1.6515, *S. baltica* NCTC-10737, *S. baltica* OS-678, *S. algae* MARS-14, and *S. benthica* KT99) and *E. coli* as the outgroup. We aligned the 16S rRNA sequences using MUSCLE (v-3.8.31) (Edgar 2004) with default parameters. Then we used PhyML (v-20120412) to construct a Maximum Likelihood tree using HKY85 model (Guindon et al. 2010; Guindon et al. 2016; Guindon 2018) with 1,000 bootstrappings. Finally, we plotted the ML tree using ggtree (Yu et al. 2017, 2018).

We analyzed syntenic relationships among the three assemblies (chromosome level) of *S. putrefaciens* ATCC-8071, CGMCC-1.6515, and CN-32. We made pairwise comparisons to obtain the synteny blocks among three assemblies using Mummer (v-4.0.0beta2) (Kurtz et al. 2004). Only alignments with over 85% coverage and more than 200 bp length were retained. The syntenic relationships among three assemblies were plotted using Circos (Krzywinski et al. 2009). We define a fragment as a rearrangement event if its upstream or downstream synteny blocks were located at the adjacent loci on the same chromosome. The dot matrix was plotted by mummerplot function of Mummer (v-4.0.0beta2).

### Comparison and Annotation of Orthologous Gene Clusters

We annotated and compared orthologous gene (OGs) clusters using OrthoVenn (v-2.0) with default parameters (Arkin et al. 2018; Xu et al. 2019). The protein sequences of five strains (*S. putrefaciens* CN-32, *S. putrefaciens* ATCC-8071, *S. putrefaciens* CGMCC-1.6515, *S. baltica* NCTC-10737, and *S. baltica* OS-678) were used as input.

### Reintroducing Functional MMR Genes of GCMCC-1.6515 into the ATCC-8071 Cells

We followed the procedures developed in Min et al. (2017). Briefly, using genome sequence and annotation of *S. putrefaciens* CGMCC-1.6515, the amplified *mutS*, *mutH*, *mutL* genes controlled by the constitutive promoter J23119 were then ligated with the pYYDT plasmid to construct pYYDT-*mutS*, pYYDT-*mutH*, and pYYDT-*mutL* (details such as primers are listed in supplementary table S25 and fig. S7, Supplementary Material online). We then transformed these plasmids into the donor strain *E. coli* WM3064 (100 μg/ml diaminopimelic acid and 50 μg/ml kanamycin were added for screening transformants) and then transferred them separately into the ATCC-8071 cells by conjugation. Finally, we performed fluctuation tests on these complemented strains, the original ATCC-8071 and CGMCC-1.6515 strains.

## Supplementary Material

Supplementary data are available at *Genome Biology and Evolution* online.

## Supplementary Material

evab148_Supplementary_Data
