# Supplementary material for: Unexpected Discovery of Hypermutator Phenotype Sounds the Alarm for Quality Control Strains
Source: Genome Biol Evol. 2021 Jun 28;13(8):evab148. doi: 10.1093/gbe/evab148 (PMC8350357; doi:10.1093/gbe/evab148)
Supplement: evab148_Supplementary_Data [file evab148_supplementary_data.zip › Supplementary Figures.pdf]

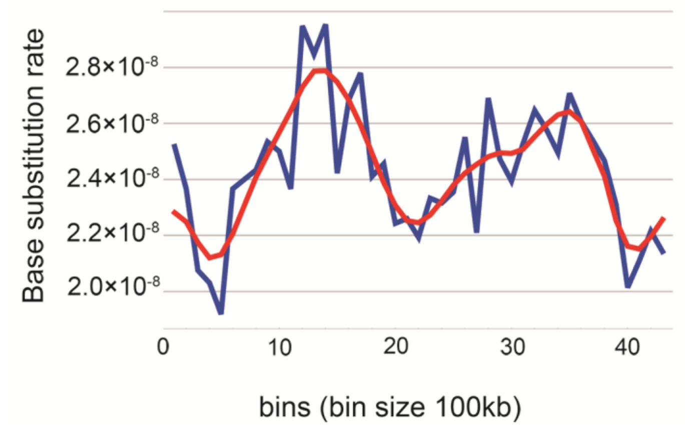

Supplemental Figure S1. The distribution of mutations along the whole-genome of ATCC-8071, which was divided into 43 equal bins. The blue curve is the mutation rate in each bin; the red curve is the value after wavelet transformation and GC content normalization (Long, et al. 2015b).

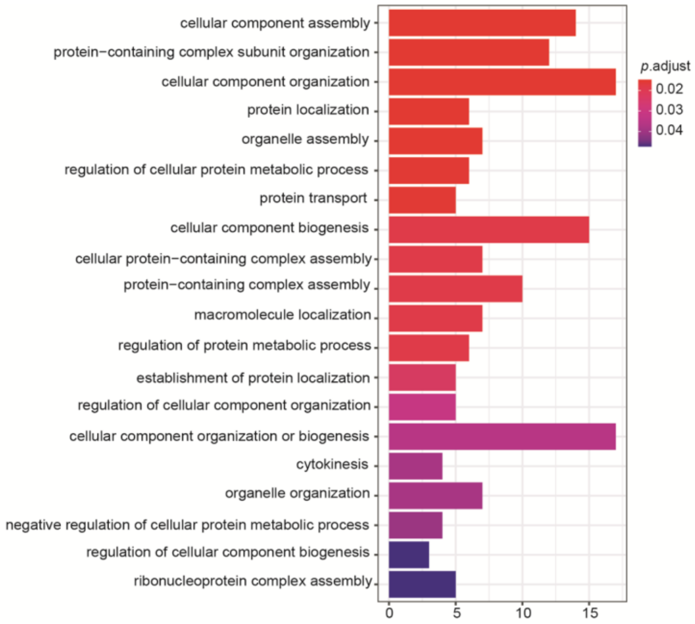

Supplemental Figure S2. The GO analysis results for genes without mutation hits in ATCC-8071 MA lines.

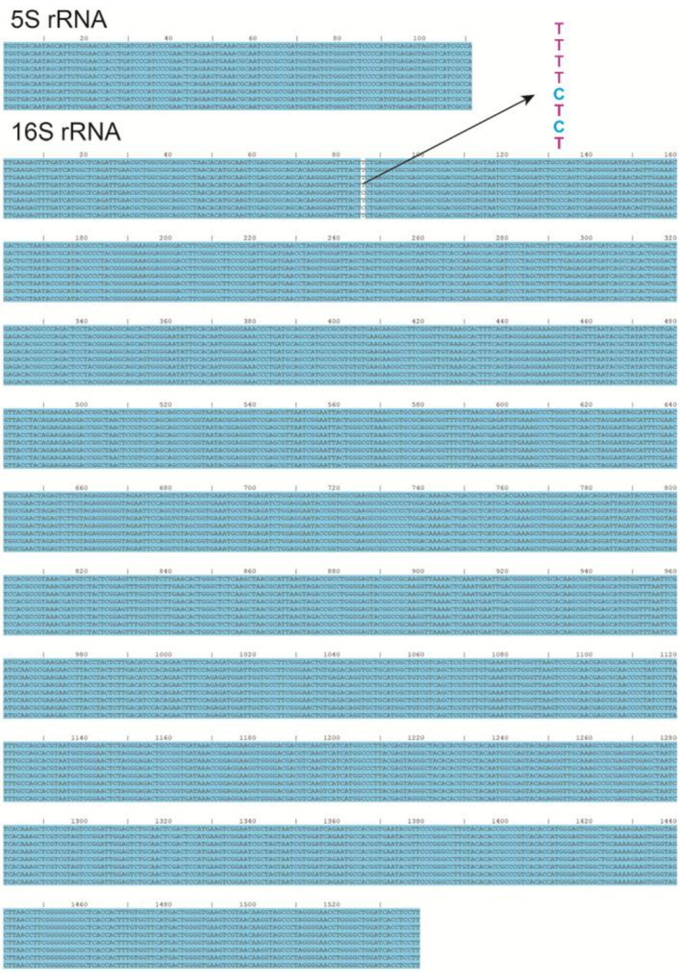

Supplemental Figure S3. The alignments of 5S and 16S rRNA genes of different rRNA operons of ATCC-8071. From top to bottom, the coordinates (start:end) of the nine 5S rRNAs are: 43,538:43,648, 222,000:222,110, 390,156:390,266, 390,458:390,568, 1,039,224:1,039,334, 2,620,348:2,620,458, 4,075,424:4,075,534, 4,144,483:4,144,593 and 4,344,534:4,344,644 respectively; the coordinates (start:end) of the eight 16S rRNAs: 38,600:40,138, 217,014:218,552, 384,598:386,136, 1,034,185:1,035,723, 2,624,382:2,625,920, 4,079,554:4,081,092, 4,148,041:4,149,579, 4,348,195:4,349,733 respectively.

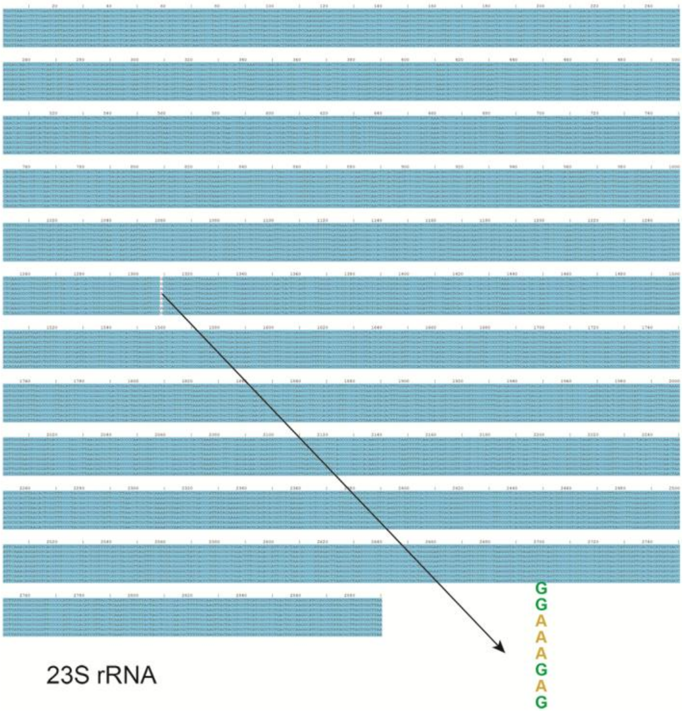

Supplemental Figure S4. The alignment of 23S rRNA genes of different rRNA operons of ATCC-8071. From top to bottom, the coordinates (start:end) of the eight 23S rRNAs: 40,468:43,357, 218,930:221,819, 387,086:389,975, 1,036,053:1,038,942, 2,620,695:2,623,584, 4,075,715:4,078,604, 4,144,774:4,147,663, 4,344,825:4,347,714 respectively.

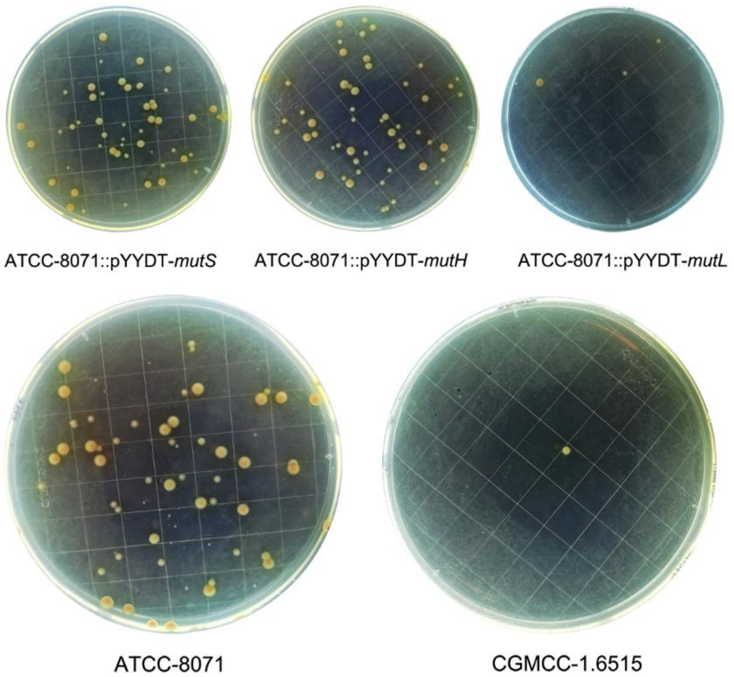

Supplemental Figure S5. The rifampicin-resistant mutants of fluctuation tests on the three strains constructed in the complementation experiments, the original ATCC-8071, and CGMCC-1.6515. ~2.27×10^8 cells were initially plated for ATCC-8071::pYYDT-*mutS*, ATCC-8071::pYYDT-*mutH*, ATCC-8071::pYYDT-*mutL*, ATCC-8071; and 4.49×10^8 cells for CGMCC-1.6515.

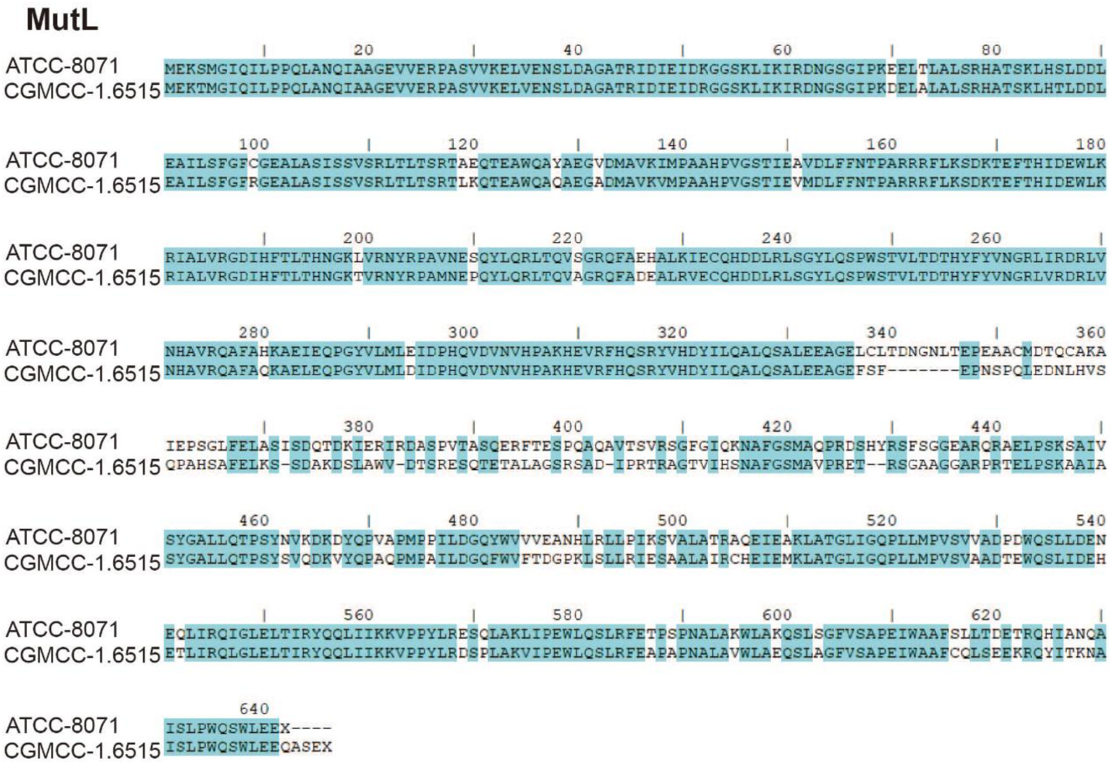

Supplemental Figure S6. The amino acid alignment of MutLs of ATCC-8071 (hypermutator) vs. CGMCC-1.6515. The divergence at the end of the alignment was taken as one indel. Xs mark stop codons.

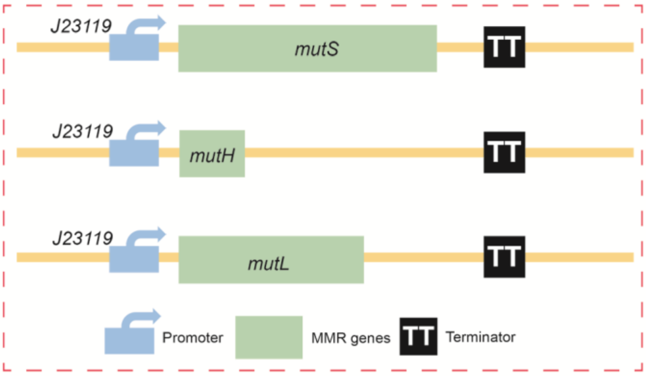

Supplemental Figure S7. Constructs for the complementation experiments. J23119 is the constitutive promoter.
